# Pay-it-forward gonorrhea and chlamydia testing among men who have sex with men and male STD patients in China: the PIONEER pragmatic, cluster randomized controlled trial protocol

**DOI:** 10.1186/s12889-023-16095-8

**Published:** 2023-06-20

**Authors:** Gifty Marley, Rayner Kay Jin Tan, Dan Wu, Tong Wang, Murong Sun, Qilei Sheng, Margaret Elizabeth Holly, Takhona Grace Hlatshwako, Cheng Wang, Weiming Tang, Rohit Ramaswamy, Ligang Yang, Danyang Luo, Sean S. Sylvia, Kurt Gray, David Van Duin, Heping Zheng, Joseph D. Tucker

**Affiliations:** 1 University of North Carolina Project-China, Guangzhou, China; 2 Clinical Research Department, London School of Hygiene and Tropical Medicine, London, UK; 3 University of North Carolina at Chapel-Hill, Chapel Hill, NC USA; 4 Dermatology Hospital, Southern Medical University, Guangzhou, China; 5 Department of Medicine, University of North Carolina at Chapel-Hill, Chapel Hill, NC USA; 6 Cincinnati Children’s Hospital Medical Center, Cincinnati, OH USA

**Keywords:** Pay-it-forward, Gonorrhea, Chlamydia, Upstream reciprocity, Warm-glow, Social innovation

## Abstract

**Background:**

Gonorrhea and chlamydia are the most common sexually transmitted diseases (STDs) among men who have sex with men (MSM) in China. Previous studies have shown pay-it-forward (PIF) interventions to be associated with a substantial increase in gonorrhea and chlamydia test uptake compared to standard-of-care. We propose a 'pay-it-forward' gonorrhea and chlamydia testing randomized controlled trial (PIONEER). The trial would evaluate the effectiveness of two pay-it-forward strategies in promoting testing uptake compared to the standard of care (in which men pay for their tests out-of-pocket) among MSM and male STD patients in China.

**Methods:**

PIONEER will be a three-armed, pragmatic cluster randomized controlled trial (RCT), conducted across 12 clinics (six MSM-led and six public STD clinics) to compare the effectiveness of three implementation strategies. Each facility will be randomized to a standard pay-it-forward intervention of gonorrhea/ chlamydia testing with minimal encouragement for testing, a community-engaged pay-it-forward arm, or a control arm where men pay for their tests out-of-pockets. The primary outcome will be dual gonorrhea/chlamydia test uptake. Secondary outcomes will include syphilis testing, amount donated in pay-it-forward, number of positive gonorrhea and chlamydia tests, and measures of antimicrobial resistance. A sequential transformative mixed methods design will be used to evaluate the implementation process in type 2 effectiveness-implementation hybrid design. Data sources will include survey on acceptability, and feelings and attitudes towards the interventions among participants; testing and treatment uptake data from clinic records, WeChat records, and qualitative data to gain insights into men's perceptions and attitudes towards the pay-it-forward, mechanisms driving uptake, and donating behaviors. Implementers and organizers will be interviewed about fidelity and adherence to protocol, sustainability of pay-it-forward intervention, and barriers and facilitators of implementing the intervention.

**Discussion:**

PIONEER will substantially increase gonorrhea/chlamydia testing among MSM in China, providing an innovative and new financial mechanism to sustain STD screening among sexual minorities in low- and middle-income countries. This study will answer compelling scientific questions about how best to implement pay-it-forward and the individual and organizational characteristics that moderate it.

**Trial registration**: The study with identification number NCT05723263 has been registered on clinicaltrials.gov/.

## Contributions to the literature

- This cluster randomized controlled trial examines the effectiveness of two implementation strategies involving different levels of community engagement to translate the generosity created by the pay-it-forward approach into test-taking action.
- A sequential transformative mixed methods design will be used to evaluate the implementation process to determine mechanisms by which pay-it-forward motivates testing and donations across the two intervention arms and how individual and organizational characteristics moderate these. 
- This study will answer compelling scientific questions about how best to implement pay-it-forward and the individual and organizational characteristics that moderate it.

## Background

Gonorrhea and chlamydia are among the four most common sexually transmitted diseases (STDs) worldwide and in China [1–5]. The World Health Organization estimated 87 million new cases of gonorrhea and 127 million cases of chlamydia among people 15–49 years old based on a global systematic review [5, 6]. The prevalence of gonorrhea and chlamydia among men who have sex with men (MSM) in southern China has been reported as high as 12.5% and 18.2% respectively [7–10].

Gonorrhea and chlamydia infection are often asymptomatic at extragenital sites [11, 12]. Hence, testing rates remain low among most high-risk populations, including MSM. These low testing rates pose a public health concern since gonorrhea and chlamydia infection are known to increase the risk of HIV acquisition [13] and United States Centers for Disease Control and Prevention guidelines [14] recommend routine gonorrhea and chlamydia testing for sexually active MSM. Studies in different provinces of China consistently report that less than half of MSM have ever been tested for gonorrhea and chlamydia [15, 16]. Low testing rates are problematic because this allows onward gonorrhea and chlamydia transmission.

Low testing rates are likely related to fees and minimal community engagement. User fees are common in many low- and middle-income countries (LMICs) for STD testing and discourage test uptake, [16] decreasing opportunities for prompt treatment and public health interventions [6, 15]. Gonorrhea and chlamydia testing in China are expensive relative to local incomes (approximately USD 20 per test) and not covered by health insurance or other social support systems [17]. This decreases access to and uptake of routine gonorrhea and chlamydia testing [16, 18]. Community engagement strategies have been an effective tool to increase access in other settings [19], yet community engagement in STD testing in China is weak [20]. Many MSM-focused campaigns for STD testing are driven by public health authorities and have limited authentic input from local end-users [21]. This limited participation may then diminish STD test uptake. In response to these financial and community barriers to STD testing, our team developed a pay-it-forward approach to gonorrhea and chlamydia testing for MSM. Additionally, gonococcal antimicrobial resistance in China is a major problem [22], with a national surveillance study among 3,849 isolates showing that 18.6% of isolates were resistant to azithromycin [23]. Expanding gonorrhea screening among MSM could prevent onward transmission, delaying the development of antibiotic resistance. However, several ecological studies have suggested widespread gonorrhea testing may lead to more antibiotic use and increased antibiotic resistance [24, 25]. Expanding gonorrhea testing within the high intensification arm of the trial will provide a unique environment for early gonorrhea detection and treatment.

**Fig. 1:**
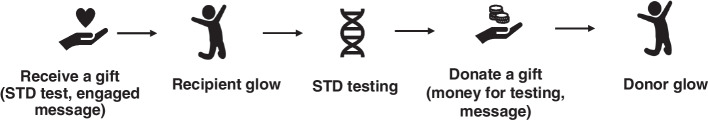
Overview of pay-it-forward. The figure summarizes the process underlying the concept of the pay-it-forward concept to be adapted in PIONEER

Pay-it-forward is a strategy to increase uptake based on a social innovation [26]. In pay-it-forward, a person receives a free STD test that is donated by the local community and is offered a chance to donate money to support testing for someone else in the future (Fig. 1) [27, 28].

Pay-it-forward chains of giving are sometimes driven by unconnected generous individuals, [28] but more often organized by a group with a common purpose [29, 30]. Studies have shown that pay-it-forward can be sustainable and promote generosity [31].

Our previous pay-it-forward research showed that the intervention was associated with a substantial increase in gonorrhea and chlamydia test uptake compared to standard-of-care [32, 33]. However, there have been several limitations. First, past projects were mostly implemented only at MSM-led clinics and not public STD clinics. While MSM-led clinics are better positioned to organize MSM community engagement activities, [20] this approach may miss out on MSM who prefer to attend public STD clinics. Second, MSM included in these studies were recruited from clinics in a central urban location [32, 33]. The pay-it-forward intervention needs to be evaluated in additional cities and different types of clinics (i.e., both MSM-led and public STD clinics).

Given that our previous pay-it-forward research focused on the efficacy of pay-it-forward, more implementation research is needed to ensure the impact of this innovation. Lack of implementation evaluation limits our understanding of the implementation process, and community-engaged strategies are needed to maximize efficiency and effectiveness. These underscore the importance of implementation research to inform contextually appropriate strategies to implement pay-it-forward most effectively.

We propose the 'pay-it-forward gonorrhea and chlamydia testing pragmatic, cluster randomized controlled trial (PIONEER)' to examine the effectiveness of two intervention strategies involving different levels of community engagement. That is to translate the generosity created by the pay-it-forward approach into test-taking action and voluntary donations. In the standard approach, men receive the gift of a test and view messages promoting testing. In the community-engaged approach, men receive gifts, view tailored messages, are encouraged to create tailored messages of their own, and are invited to become part of a community of collaborators engaged in promoting gonorrhea/chlamydia testing. Community-engaged approaches are important for enhancing the contextual appropriateness of public health interventions and the possibility of success [34].

This study is a Type 2 effectiveness-implementation hybrid design with two coprimary aims of determining the effectiveness of clinical interventions and the feasibility and potential utility of an implementation intervention or strategy [35]. The co-primary aims are as follows:

1. To compare the primary outcome of gonorrhea/chlamydia test uptake and secondary outcomes like amount donated (among those in the pay-it-forward arms), antimicrobial resistance measures, positive tests for gonorrhea and chlamydia, gratitude measures, and community solidarity measures in a standard pay-it-forward arm, a community-engaged pay-it-forward arm and a control arm using a three-arm cluster randomized controlled trial.
2. To determine how pay-it-forward motivates testing and donations across the two intervention arms and how individual and organizational characteristics moderate these.

### Theories informing the PIONEER intervention

Our trial design and pay-it-forward strategies were informed by two theories related to gift giving and social reciprocity. First, Marcel Mauss and others have developed substantial theoretical and empirical literature describing gift-giving's expressive and instrumental function in specific social contexts [36, 37]. Second, the pay-it-forward intervention is a novel concept embedded in the upstream reciprocity theory [38]. Social reciprocity provides a framework to understand how STD testing could be normalized within local social relationships. The idea is that if someone is generous to you, it generates a 'recipient glow' that encourages you to be generous to others. In the context of pay-it-forward, the hypothesis is that someone receiving a gift feels obligated to be generous to the community by getting tested and contributing a gift in return.

### Principles guiding the design of community engagement elements

Our implementation strategies were developed based on co-creation approaches that engaged members of the target population [39]. Community participation is essential to an intervention's acceptance and contextual appropriateness [34]. This is especially important for hard-to-reach sexual minority groups. Co-creation approaches involve collaboration between researchers and community members that involve joint decision-making on research goals, processes, and outcomes [40]. Evidence has shown that community engagement in developing intervention strategies is associated with better acceptance, adherence, and effectiveness of the interventions in changing behaviors in a good way [41–43]. We have adopted co-creation approaches to guide the following activities: 1) organizing a co-creation group to co-create the community-engaged pay-it-forward strategy and forming shared leadership; 2) guiding the design of community engagement elements of the community-engaged strategy; 3) involving key stakeholders in various stages of the project to enhance appropriateness, acceptability, and feasibility.

## Methods

### Design overview

Our trial will use a type 2 effectiveness-implementation hybrid study design that values intervention effectiveness and implementation process. Specifically, the PIONEER trial will be a three-arm, cluster randomized controlled trial (RCT), followed by a mixed-methods implementation evaluation study. The cluster RCT aims to compare the effectiveness of the three implementation strategies, and the implementation evaluation will investigate other domains of the implementation process guided by the RE-AIM framework. The study has been registered on clinicaltrials.gov with identification number: NCT05723263.

The cluster RCT will enroll 12 diverse clinics (i.e., six MSM-led and six public STD clinics) and stratify the randomization by clinic type. The clinics will be assigned to one of the three study arms: a standard pay-it-forward arm with minimal encouragement to get tested, a community-engaged pay-it-forward strategy, and a control arm in which men pay for their tests out-of-pocket. The standard pay-it-forward strategy, developed through our previous pay-it-forward research [33], will have men who enter the clinic receive a free gonorrhea/chlamydia test alongside handwritten postcards from other MSM in the local community. The community-engaged strategy will include all the standard pay-it-forward components with additional community engagement activities. The gift recipients will be invited to submit handwritten postcards of their own, print images from instant cameras, or create videos promoting gonorrhea/chlamydia testing (to be viewed at the same clinic). Men who agree to participate will be invited to a hybrid digital and in-person pay-it-forward event where they meet other people who have donated and to related participatory activities organized by the local clinic to promote gonorrhea and chlamydia testing.

Mixed methods will be used to evaluate the implementation process with project implementers, organizers, and participants. Data will be obtained from multiple sources, including an online survey on participants' acceptability, appropriateness, feelings, and attitudes towards pay-it-forward as an intervention. We will also collate administrative data about test uptake, treatment rate, and qualitative data to gain insights into men's perceptions and attitudes towards the pay-it-forward strategies and the mechanisms driving uptake. Surveys and qualitative interviews will be conducted with implementers and organizers about fidelity and adherence to protocol, intention to continue and maintain a pay-it-forward intervention, and barriers and facilitators of implementation.

### Study setting and population

We will implement the study in the following Guangdong cities: Zhuhai, Foshan, Jiangmen Shenzhen, Zhanjiang, Dongguan, and Yunfu. These clinics were chosen because their respective cities have a higher burden of STDs, and findings would be potentially relevant in many cities.

The study settings between public STD clinics and MSM-led clinics would be different. One key obstacle to recruiting MSM in public STD clinics is that MSM is the risk of unintentional sexual identity disclosure due to justifiable fears about risks of stigma and discrimination from other men and health workers. Recruitment in public STD clinics will be extended to include all men visiting the public STD clinic regardless of sexual orientation to avoid the risk of unintentional disclosure. This will also lower the risk of MSM patients feeling and being unnecessarily 'targeted.'

Eligibility criteria for recruitment at public STD clinics include all men (regardless of sexual orientation) that are 1) 18 years and above; 2) have had sex over the past year; 3) have not tested for gonorrhea and chlamydia in the past year; 4) resided in the city in the past three months; 5) speak Mandarin or Cantonese, and 6) mentally capable of providing informed consent for gonorrhea and chlamydia testing. Only MSM recruited through this method will be included in the analytic sample. Data from non-MSM participants will be analyzed in a separate sub-study.

Inclusion criteria for recruitment at MSM-led clinics include all men who had anal sex with other men that are 1) at least 18 years old; 2) have had anal sex over the past year; 3) have not been tested for gonorrhea and chlamydia in the past year; 4) have resided in the city in the past three months; 5) speak Mandarin Chinese or Cantonese, and 6) mentally capable of providing informed consent to test for gonorrhea and chlamydia.

### Development of intervention and formative research

#### Approach and study team

A key innovation of this trial study, relative to past pay-it-forward research, is developing and evaluating the community-engaged approach as an additional intervention arm. While upstream reciprocity is hypothesized to promote the pay-it-forward mechanism, simulation models of the process show that the "warm glow" does not guarantee sustainability [44]. In contrast, evidence shows that when combined with network reciprocity, where acts of altruism are reinforced by a community of collaborators, the probability of cooperation increases [45]. The two arms of our trial are designed to test these two hypotheses.

The study will be managed through a consortium involving two academic centers (the University of North Carolina in Chapel Hill and the Southern Medical University Dermatology Hospital in Guangzhou). The study implementation will be undertaken in partnership with a non-profit CBO that will organize community engagement activities (the Social Entrepreneurship to Spur Health 'SESH'). The SESH team has a strong track record of community engagement and organizing research studies on sexual health, including pay-it-forward programs in Beijing and Guangzhou. The study team would comprise academic and SESH staff with expertise in community mobilization, epidemiology, implementation science, health economics, health statistics, and social science.

A co-creation group will be appointed and would comprise MSM representatives, clinicians, and nurses, a communication officer co-chaired by members of SESH, and a local MSM community-based organization. Co-creation groups serve as a source of shared leadership in community-led research, and their members typically reflect the community of interest. The co-creation group will be consulted quarterly to provide input on the study design, implementation, and dissemination activities.

#### Site selection, training, and trial registration

We will implement the study in Guangdong cities with MSM-led clinics: Guangzhou, Shenzhen, Zhuhai, Jiangmen, Foshan, and Dongguan. These clinics were chosen because respective cities have a higher burden of STDs, they include the two major service delivery approaches (MSM-led clinics and public STD clinics) in China, and findings would be potentially relevant in many cities. Each selected clinic has a dual chlamydia/gonorrhea point-of-care testing service and a stable supply chain of ceftriaxone and azithromycin. Each site will receive Good Clinical Practice (GCP) training for new personnel, regulatory assessment, and related trial requirements. A representative from each participating clinic will be invited to attend a single training workshop in Guangzhou to introduce the study protocol, local implementation workflows, gonorrhea and chlamydia treatment, and resistance testing processes. We will register the RCT on ClinicalTrials.gov prior to the commencement of the trial.

#### Developing a standard strategy and the community-engaged strategy

This three-arm cluster randomized controlled trial will compare gonorrhea and chlamydia test uptake in a standard pay-it-forward implementation strategy arm, a community-engaged pay-it-forward implementation strategy arm, and a control arm. Our implementation strategies are summarized in Table 1.

**Table 1:** Overview of the three study arms

Trial Arm	Financial Component of Gonorrhea/Chlamydia Testing*	Community Engagement
*Standard Pay-it-forward arm*	Free gonorrhea/chlamydia testing for all recruited participants in both the MSM-led and public STD clinics	Passive: viewing postcards and materials written by others encouraging gonorrhea/chlamydia testing
*Community-engaged Pay-it-forward arm*	Free point-of-care gonorrhea/chlamydia testing for all recruited participants in both the MSM-led and public STD clinics	Active: multi-stakeholder co-creation activities to develop essential components of the intervention and implementation strategies; writing postcards; opportunity to donate to support others
*Control arm*	Fee-based point-of-care gonorrhea/chlamydia testing	None

In the standard pay-it-forward arm, all recruited participants in the MSM-led and public STD clinics will be given a donated free test alongside handwritten postcards from other participants in the local community. Standard operating procedures, survey instruments, and educational materials developed in our previous RCT will guide this approach. The postcards will be generated through an open call for suggestions about tailored, hand-written messages that are clinic specific and locally appropriate. We will use standardized methods developed by the World Health Organization to design the open call [46, 47]. This will involve creating a steering committee, promoting the open call, judging submissions, recognizing excellent submissions, and implementing selected ideas.

The community-engaged arm will include the standard pay-it-forward strategy with key community-engagement activities. Gift recipients will be invited to submit handwritten postcards or create videos promoting gonorrhea and chlamydia testing (to be viewed at the same clinic). Men who agree to participate will be invited to a pay-it-forward event to meet other people who have donated. They will also be invited to related activities organized by the local clinic to promote gonorrhea and chlamydia testing.

In the control arm, participants will be informed about the importance of gonorrhea and chlamydia testing but will not receive handwritten postcards or other community engagement activities. Among men who choose to receive gonorrhea and chlamydia testing, they will have access to the same diagnostics, treatment, and follow-up provided in the other two arms. Donations in the two pay-it-forward arms would be received and tracked using WeChat, a hybrid social media app in China that allows micro-payments. Each clinic will have its own WeChat account for donations.

### Pragmatic cluster RCT

#### Sample size and power considerations

We used a binary outcome (uptake of gonorrhea/chlamydia test) cluster RCT design for sample size calculation where the unit of randomization is the clinic. In order to achieve a 90% power and allow for a 0.05 type-I error, 12 overall clusters (4 in each arm) are needed. Thus, the sample size includes 12 clusters and 1200 participants (100 per cluster), estimated based on the sample size calculation principles for cluster randomized trials. We expect this study will have a 90% power to detect a 10% difference in azithromycin resistance between intervention arms and routine gonorrhea and chlamydia antibiotic surveillance data from other cities in the same province. Nevertheless, further calculations will be made following formative research endeavors that will estimate the prevalence of these outcomes. The calculation would be performed using the software PASS (version 15) with the formulas developed by Hussey and colleagues [48].

#### Randomization

**Fig.2:**
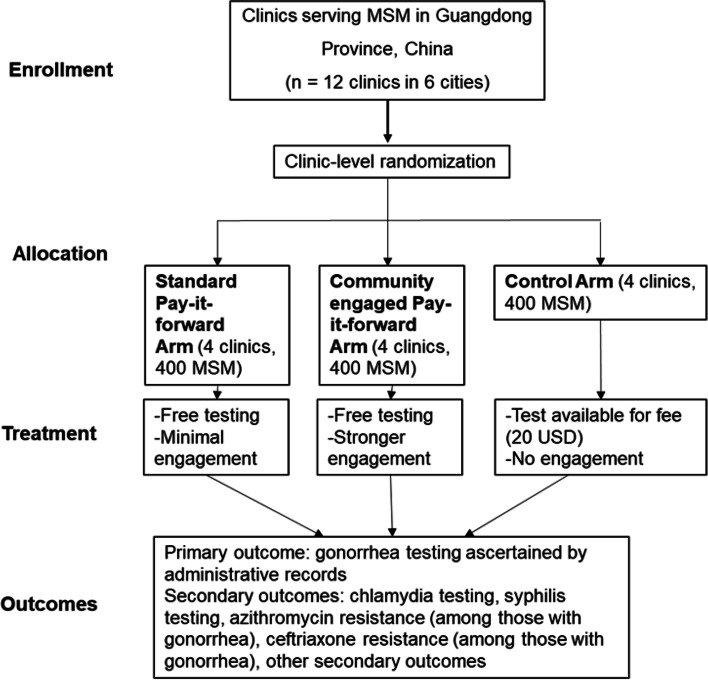
Pragmatic Cluster RCT design flowchart. The figure shows the process of study sites distribution, participant enrollment and participant randomization for the cluster RCT. Each city will have one MSM-led clinic and one public clinic participating

Figure 2 shows the cluster RCT design flowchart. We will assign clinics to study arms on a 1:1:1 basis using covariate constrained randomization. We will use covariate constrained randomization to decrease potential bias associated with unbalanced study arms. We will use block randomization to ensure the same number of MSM-led and public STD clinics in each arm because these are unique implementation structures. All men recruited via one clinic will be assigned to one arm. We will recruit and screen men until we meet our sample size of 1200 men.

#### Intervention delivery strategies

The study duration is estimated to be up to one year at each clinic, with the potential to continue based on local commitments. All clinics will use standard gonorrhea/chlamydia molecular tests approved by the US FDA and the Chinese national regulatory authorities. Men who agree to be tested will have specimens collected from pharyngeal, rectal, and urethral sites. Specimens will be pooled based on excellent sensitivity and specificity. The turnaround time for test results will depend on the local clinic. Surveys will be self-administered online to facilitate implementation. All participants diagnosed with gonorrhea or chlamydia will receive antibiotic treatment per the clinical protocol in China.

#### Outcome measures

The primary outcome of the RCT will be gonorrhea and chlamydia test uptake in the pay-it-forward arms (both standard and community-led) compared to the control arm ascertained by clinical records. Secondary outcomes will include syphilis testing, amount donated towards pay-it-forward, antimicrobial resistance measures, positive tests for gonorrhea and chlamydia, measures of gratitude and community solidarity, and per unit costs associated with the intervention (Table 2).

**Table 2:** Secondary outcomes of the cluster RCT

*Outcome*	*Time Frame*	*Ascertainment*
Syphilis testing	During enrollment visit	Administrative data
Amount donated	During enrollment visit	Administrative data
Azithromycin resistance based on culture and molecular methods	During enrollment visit	Laboratory result
Ceftriaxone resistance based on culture and molecular methods	During enrollment visit	Laboratory result
The number of participants who tested positive for Gonorrhea	During enrollment visit	Laboratory result
The number of participants tested positive for chlamydia	During enrollment visit	Laboratory result
Cost per test^a^	During enrollment visit	Costing data collection
Adapted gratitude scale	During enrollment visit	Self-report
Community solidarity scale^b^ [49]	During enrollment visit	Self-report^2^

^a^Cost per test is defined as the cost associated with respective interventions (development, start-up, implementation, intervention) per individual who reported testing for gonorrhea and chlamydia following the intervention; economic test and financial cost are reported separately

^b^Community solidarity involves engagement, social network support, and a sense of belonging. These items were used in our previous pay-it-forward research study [49]

No randomized controlled trials have examined the relationship between more intensified gonorrhea/chlamydia testing and the development of antibiotic resistance because of more prompt treatment. We have therefore included antimicrobial resistance measures as secondary outcomes.

#### Data analysis

The primary outcome will be gonorrhea/chlamydia test uptake among MSM. Generalized mixed-effects models will be used to examine the two main hypotheses: 1) comparing the superiority of the community-engaged strategy to the standard pay-it-forward strategy and control arm, and 2) comparing the superiority of the standard pay-it-forward strategy to the control arm. The estimated intervention effects will be reported with 95% CIs and p-values. Descriptive analysis will be used to summarize the characteristics and behaviors of the participants in each arm.

We will include participants diagnosed with gonorrhea and chlamydia whose isolates can be cultured and calculate the minimum inhibitory concentrations (MICs) for specific antibiotics. MICs will be described as geometric means, and the distributions among gonorrhea/chlamydia strains in the intervention arm will be compared to surveillance data using a Mann–Whitney test. We will use the midpoint between the specific value and the next higher or lower dilution value to calculate the mean for MICs above or below the pre-specified thresholds. We will calculate the prevalence rates and 95% confidence intervals of gonorrhea/chlamydia resistance and decreased susceptibility to pre-specified antibiotics. Bivariate analyses and multinomial logistic regression will be used to determine the variables associated with infection with resistant or decreased susceptible strains of antibiotics. We will compare the frequency of resistant strains in the three arms using a chi-squared test for trend.

#### Oxytocin sub-study

One community-engaged MSM-led clinic and one control MSM-led clinic will be selected to conduct a pilot quasi-experimental study to assess the impact of the community-engaged intervention on measures of salivary oxytocin. We aim to recruit 100 participants from each site (*n* = 200) for this sub-study. This sub-study aims to investigate the use of oxytocin as an objective biomarker to reflect feelings of warm glow. Oxytocin is a hormone produced in the hypothalamus and associated with trust and altruism measures [50–52]. Studies have also administered intranasal oxytocin to study its effect on gratitude and pro-social actions [51, 52]. We aim to measure salivary oxytocin before and after the intervention to ascertain if such changes may be attributable to the intervention. Changes in oxytocin levels will also be measured against changes in levels of warm glow and other baseline characteristics to ascertain the relationship between these factors. Collected saliva samples will be stored at -40 degree celsius for a minimum of 6 months after collection and disposed of following the standard laboratory protocol per study site.

### Implementation evaluation

The RE-AIM framework has been extensively used in implementation research to evaluate the implementation of evidence-based interventions [53]. The RE-AIM framework is one of the most commonly used frameworks to evaluate the implementation of an intervention. It guides the development of implementation-focused outcomes to understand better mechanisms and factors influencing behavioral changes due to interventions. Evaluation of the five key domains of the implementation process will provide evidence to ensure impact of the intervention and facilitate evidence-based practices.

We will evaluate the other dimensions of implementing the standard pay-it-forward and the community-engaged pay-it-forward strategies to understand the factors influencing men to get tested and motivating their donations. Given the novelty of pay-it-forward to raise funds, its sustainability partly depends on a willingness to donate. Understanding the drivers of financial sustainability across individuals and clinics may help inform policymakers and interested researchers in resource-limited settings about the potential of using the strategy to promote underfunded public health services.

We will use a sequential transformative mixed methods study that includes semi-structured interviews and an online survey. A sequential transformative approach was selected to allow us to use a participatory action research lens more firmly [54]. In addition, this framework will help us center marginalized groups' experiences, examine inequities that have contributed to the marginalization, and link the research to actions focused on alleviating disparities [55]. Details of these instruments are provided in Table 2. In-depth interviews grounded in social reciprocity theories will explore the processes and mechanisms that affect the results and how these differ by the individual (geography and socioeconomics) and organizational (geography and readiness) characteristics. A mixed methods study will be conducted during and after the RCT.

#### Quantitative surveys

Surveys with all clinic staff involved in implementation, project implementers, and participants will be conducted through an organizational readiness survey in all selected clinics. The survey will assess the motivation and capabilities of each clinic to deliver gonorrhea and chlamydia testing and treatment services. A member of the Guangdong Provincial STD Control Center staff will assess the extent to which pay-it-forward is implemented following the protocol in each intervention clinic among a convenience sample of 10 men. All recruited participants in the standard and community-engaged pay-it-forward arms will be asked to take a brief survey assessing their perceptions of acceptability and appropriateness of pay-it-forward and the relevant implementation strategy. They will also complete a pay-it-forward-specific adapted version of the Gratitude Questionnaire-6 (GQ-6).

#### Qualitative interviews

Participants and pay-it-forward organizers will be recruited for in-depth interviews. We will recruit a purposive sample of participants in the clinic immediately following participation in the RCT in each implementation strategy arm that is representative of those who participate and those who do not, and those who gave higher and lower donations. Organizers will be recruited by sending a text message to a purposive sampling of organizers. This will include those at MSM-led and public STD clinics, organizers who identify as gay and those who do not, and other key characteristics. We will develop a topic guide on literature review, field notes, and reflections during the implementation of RCT to steer the participant interviews.

Interview topics will include (1) reasons for getting tested; (2) understanding and perception of the pay-it-forward concept; (3) feelings of warm glow after receiving a test that another community member supports; and (4) self-identity in the MSM community and level community-integration; (5) intention and motivation to initiating and mediating helping behaviors within the community. The interviewer will obtain recorded verbal informed consent from all interviewees before the commencement of the interview, which will last about one hour in person or over the phone, based on participant preference.

Interviews will be audio-recorded, but personal information will be exempted. Unintentional recording of personal information will otherwise be redacted before transcription. All audio-recorded interviews will be transcribed, and one research staff member will check the transcripts. Interview summaries will be written up for preliminary data analysis. In addition, we will collect anonymized texts from handwritten postcards and other community engagement activities, and each participant will receive a small monetary incentive of ¥50 (7.50 USD) for participation.

#### Sample size and power considerations

Twenty MSM participants and 20 pay-it-forward organizers will be recruited for individual interviews. Enrollment will cease when no new themes and sub-themes emerge, inferring data saturation. The staff of all eight participating clinics implementing the pay-it-forward intervention (both standard strategy and community-engaged strategy) will be invited to respond to questions related to implementation (see Table 2). All participating clinics will be invited to answer questions about organizational implementation and maintenance. Operational definitions and example measure of the five dimensions of RE-AIM adapted for the study are shown in Tables 2 and 3.

**Table 3:** Dimensions of RE-AIM in implementation research adapted for pay-it-forward (for MSM-led clinic sites)

Dimensions	Level	Operational definitions	Measurement method
Reach	Individual	• The proportion of MSM who enter the clinic who are screened for eligibility • Percent of MSMs who visit the clinic compared to the MSM in the catchment area	• Administrative data will be collected on the demographics, socioeconomics, and geographic location (zip code or equivalent level) of those enrolled in pay-it-forward to test if there are differences in the characteristics of those who get tested and those who do not • The population of MSMs will be collected from public data to assess the potential reach of the pay-it-forward intervention within the overall MSM population in the area
Effectiveness	Individual	• How effective are the two implementation strategies in promoting gonorrhea and chlamydia test uptake? • Does pay-it-forward influence secondary outcomes (chlamydia testing, treatment, and prevention outcomes)?	• The primary outcome is gonorrhea and chlamydia test uptake from the RCT, which will be measured using administrative data from participating clinics
Adoption	Individual	• Do the participants find pay-it-forward acceptable and appropriate? • Is there a relationship between acceptability and appropriateness and testing outcomes? • What are the demographic, socio-economic, and geographic differences between those who found pay-it-forward acceptable and appropriate and those who did not?	• Quantitative data will be collected using an adapted version of the Acceptability of Intervention Measure (AIM) and the • Intervention Appropriateness Measure (IAM). Qualitative data will be collected from interviews to gain additional insights into the results from these instruments
	Organizational	• How do feelings of social reciprocity contribute to test adoption and donation behaviors?	• The R = MC^2^ instrument will be adapted for use in clinics
Implementation	Organizational	• What is the extent to which local staff implements the pay-it-forward according to the SOP, and to what extent are legitimate adaptations needed?	• Fidelity checklists (as specified by Carroll et al.) will be used to measure adherence to protocol related to content and dose • Interviews with clinic staff will be used to determine reasons for deviation from protocol and to track and document legitimate adaptation
Maintenance^a^	Individual	• What is the average donation per participant in each arm? • Is there a relationship between testing and donations? • What are the facilitators and barriers to donation? • What are the demographic, socio-economic, and geographic differences between men who tested after receiving the gift relative to those who were not?	• The tracking of donations stratified by testing status, socioeconomics, and demographics. Interviews with participants will examine the individual-level context of donating
	Organizational	• What is the average donation per clinic in each arm? • Is organizational readiness associated with higher donation rates? • What are resource commitments from the public health sector, MSM organizations, and others to sustain pay-it-forward?	• Administrative data will be used to track donations at the clinic level • Interviews with organizers who can continue the program after the 12-month RCT phase will examine facilitators and barriers to maintaining the intervention

^a^Maintenance data will be collected for an additional 12 months (following the RCT) at selected clinics

#### Outcome measures

The outcomes will include (1) primary RE-AIM data comparing differences between the community-engaged pay-it-forward strategy and the standard pay-it-forward strategy; (2) qualitative data describing mechanisms related to test uptake and donation decisions, respectively.

#### Data analysis

Thematic analysis will be used to analyze qualitative data using a codebook developed by a senior qualitative researcher. Two research assistants will then code the transcripts based on the codebook. NVivo version 12 (Nvivo, Columbus, OH) will be used for qualitative data analysis. We will conduct both intention-to-treat (ITT) and per-protocol data analysis to assess outcomes of interest. Descriptive data analyses, independent sample t-tests, and chi-square tests will be used to compare differences in RE-AIM measures between pay-it-forward and standard-of-care arms. Multiple imputation will be used to input data for variables with > 5% missing. We will generate a global implementation score and scores of separate dimensions through evaluations against the RE-AIM framework and convert them to a z-score. Correlates of global implementation and implementation dimensions will be examined using general linear mixed models (GLMM). In addition, qualitative data on social networks will be applied to disentangle the connection between an individual's gratitude and position within a network of helping relationships (both direct and indirect relationships). Finally, we will use linguistic inquiry word count to analyze textual information gathered in community engagement activities.

#### Dissemination plan

We will communicate trial results and interpretation of our findings to the public through manuscript publications.

### Safety management plan

Criteria for withdrawal: project implementers will monitor data and safety if participants want to quit the study for any reason.

Protocol amendment: all important protocol modifications such as changes to eligibility criteria, changes to implementation procedures, new outcomes of interest, and sub-groups analyses approaches will be submitted to the relevant IRB committees for evaluation and approval before implementation. Approved changes will be communicated to project staff at implementing sites through site visits and dissemination meetings. Consortium members, co-creation groups and advisory board members will be informed during monthly update meetings and changes will be updated on the trial registration websites. Participants who have already completed the participation process will not be informed of these changes as it is a one of study.

### Data management information

All survey data will be entered into computers at the UNC Project China office as participants complete the surveys. Programs to ensure accuracy, completeness, and internal consistency are automated. Data can be readily downloaded and converted to commercially available statistical software format. During the collection of the online portion of the study, all data will be transmitted securely using SSL (TLS) 128-bit encryption across the Internet (HTTP). SSL assures users access to a valid, "non-spoofed" site and prevents data interception or tampering with sensitive information. The SSL certificate that will be used for this project will use 128-bit encryption, the preferred security level of government and financial institutions. 128-bit encryption offers virtually unbreakable protection. For example, if a hacker could crack a standard 40-bit SSL session in a day, it is estimated that it would take well beyond a trillion years to accomplish the same against a 128-bit SSL session. A dedicated server, which eliminates security issues involved with shared hosting environments where hundreds of websites and users reside on one shared web server and ensures both physical and network security, will be used to house the data. Data will then be stored in a secured server at Dermatology Hospital, Southern Medical University. The server will be configured with a redundant hard drive array to ensure reliability. Access to the data will be password protected within the server's firewall. Only the PI and a designated senior staff member will have the password to access the "key" that links the non-descript identifier to personally identifiable information. IP addresses of participants' computers will not be collected at any time. Cookies will not be used in any way to track participant activity. A quick link will exist on each survey page to give participants a fast way to switch to an innocuous website if their privacy is interrupted while completing the survey.

Data from participants' urine testing results and clinical outcomes will be recorded directly and input into Health Information System (HIS) at Dermatology Hospital, Southern Medical University. Data will be downloaded and stored in the same way as the survey data. Urine samples will be stored at -20 degree celsius for a minimum of 6 months after the collection and disposed of following the standard laboratory protocol per study site.

A data monitoring committee will not be needed for this study as there is no blinding of researchers to the randomization and allocation process. Furthermore, this trial has previously been implemented without reports of any safety concerns. Hence, we believe our internal data quality assurance systems will suffice in monitoring.

## Discussion

Pay-it-forward is a novel behavioral and financial model. Our previous studies showed that it is cost-effective compared to standard service delivery. However, the study findings have limited representativeness due to the primary involvement of MSM-led clinics but excluding public STD clinics that are major providers for STD testing in the Chinese setting. There is also a lack of understanding of the implementation process and a missed opportunity of optimizing the implementation strategy. By enhancing the community engagement components of the strategy, our trial aims to test the effectiveness of an enhanced pay-it-forward implementation strategy compared to the standard pay-it-forward approach and a control arm. We propose a programmatic cluster RCT to document high-quality evidence on the effectiveness of different pay-it-forward implementation strategies and better understand real-world practices. The proposed study will provide evidence on whether community-engaged pay-it-forward strategies can strengthen the effectiveness of pay-it-forward interventions. This would inform future expansion of pay-it-forward intervention strategies to improve gonorrhea and chlamydia testing and treatment for MSM in diverse clinic settings in China.

This study has important implications for evidence-based practices for delivering important but underfunded public health services in a novel and efficient way. Hybrid implementation study designs focus on effectiveness and assessing implementation outcomes. Generating evidence on the implementation will inform evidence-based practices and enable us to tap into the potential of normalizing or integrating the pay-it-forward interventions into existing programs to promote gonorrhea/chlamydia testing among a key community. Our hybrid type 2 study design tests two pay-it-forward implementation strategies and evaluates other domains, including reach, adoption, implementation, and maintenance. These will help inform what is possible in routine practices, translate researching findings into practices and assess the strategy in gonorrhea/chlamydia testing service delivery.

Our trial will also add value to the current literature on generosity and reciprocity and how they may change behaviors. Our implementation evaluation will underscore the individual and organizational-level mechanisms affecting test uptake and donating behaviors in the pay-it-forward arms. We will test against the upstream reciprocity theory and measure whether generosity is associated with uptake and donating behaviors. This evidence will be relevant to interested health researchers, program implementers, and public health authorities to leverage human kindness and a 'warm glow' to promote healthy behaviors and engage key stakeholders.

We anticipate that this novel financing model may also have policy implications for transitioning fee-based to subsidized health services in China and beyond. Pay-it-forward is effective in pooling small funds for essential services to support other service users in the community. Such pro-social behaviors could potentially be contagious when managed well. If proven practically more cost-effective, pay-it-forward strategies could help mobilize micro-donations from economically better-off areas to cover essential health services in underdeveloped places.

## Data Availability

All investigators and implementation staff will have unrestricted access to the full data set for verification interpretation purposes. Data will be made available to the public after publication of study findings upon request from the corresponding author.

## Abbreviations

CBO: Community Based organization
GLMM: General Linear Mixed Models
HTTP: Hypertext Transfer Protocol
LMICs: Lower/Middle Income Countries
MSM: Men who have Sex with Men
PIF: Pay-It-Forward
RCT: Randomized Controlled Trial
SSL: Secure Sockets Layer
STD: Sexually Transmitted Disease
TSL: Transport Layer Security
USD: United States dollar
WHO: World Health Organization

## References

[1] Kirkcaldy RD, Weston E, Segurado AC, Hughes G (2019). Epidemiology of gonorrhoea: a global perspective. Sex Health.

[2] Ye X, Liu J, Yi Z (2019). Trends in the Epidemiology of Sexually Transmitted Disease, Acquired Immune Deficiency Syndrome (AIDS), Gonorrhea, and Syphilis, in the 31 Provinces of Mainland China. Med Sci Monit.

[3] Wi T, Lahra MM, Ndowa F, Bala M, Dillon J-AR, Ramon-Pardo P (2017). Antimicrobial resistance in Neisseria gonorrhoeae: global surveillance and a call for international collaborative action. PLoS Medicine.

[4] Wang C, Tang W, Zhao P, Tucker J, Chen L, Smith MK (2019). Rapid increase of gonorrhoea cases in Guangdong Province, China, 2014–2017: a review of surveillance data. BMJ Open.

[5] Rowley J, Vander Hoorn S, Korenromp E, Low N, Unemo M, Abu-Raddad LJ (2019). Chlamydia, gonorrhoea, trichomoniasis and syphilis: global prevalence and incidence estimates, 2016. Bull World Health Organ.

[6] World Health Organization (2016). Global Health Sector Strategy on Sexually Transmitted Infections 2016–2021.

[7] Chen X-S, Peeling RW, Yin Y-P, Mabey DC (2011). The epidemic of sexually transmitted infections in China: implications for control and future perspectives. BMC Med.

[8] Yang LG, Zhang XH, Zhao PZ, Chen ZY, Ke WJ, Ren XQ (2018). Gonorrhea and chlamydia prevalence in different anatomical sites among men who have sex with men: a cross-sectional study in Guangzhou, China. BMC Infect Dis.

[9] Lin XX, Meng SY, Ke WJ, Zhang XH, Wang LY, Liao YY (2022). Community engagement on-site rapid test for chlamydia and gonorrhea among men who have sex with men: a pioneering study in Guangzhou, China. BMC Public Health.

[10] Zhou Y, Cai YM, Li SL, Cao NX, Zhu XF, Wang F (2019). Anatomical site prevalence and genotypes of Chlamydia trachomatis infections among men who have sex with men: a multi-site study in China. BMC Infect Dis.

[11] Detels R, Green AM, Klausner JD, Katzenstein D, Gaydos C, Handsfield HH (2011). The Incidence and Correlates of Symptomatic and Asymptomatic Chlamydia trachomatis and Neisseria gonorrhoeae Infections in Selected Populations in Five Countries. Sex Transm Dis.

[12] Lutz AR (2015). Screening for Asymptomatic Extragenital Gonorrhea and Chlamydia in Men Who Have Sex with Men: Significance, Recommendations, and Options for Overcoming Barriers to Testing. LGBT Health.

[13] World Health Organization (2011). Prevention and Treatment of HIV and Other Sexually Transmitted Infections Among Men Who Have Sex With Men and Transgender People: Recommendation for a Public Health Approach Geneva, Switzerland WHO Document Production Services.

[14] CDC. 2015 sexually transmitted diseases treatment guidelines 2015. Available from: https://www.cdc.gov/std/tg2015/screening-recommendations.htm.

[15] Wu D, Li KT, Tang W, Ong JJ, Huang W, Fu H (2019). Low chlamydia and gonorrhea testing rates among men who have sex with men in Guangdong and Shandong Provinces, China. Sex Transm Dis.

[16] Lin L, Nehl EJ, Tran A, He N, Zheng T, Wong FY (2014). Sexually transmitted infection testing practices among ‘money boys’ and general men who have sex with men in Shanghai, China: objective versus self-reported status. Sexual health.

[17] Zhang X, Nie H (2021). Public health insurance and pharmaceutical innovation: Evidence from China. J Dev Econ.

[18] Zheng N, Guo Y, Padmadas S, Wang B, Wu Z (2014). The increase of sexually transmitted infections calls for simultaneous preventive intervention for more effectively containing HIV epidemics in China. BJOG.

[19] Zhang TP, Liu C, Han L, Tang W, Mao J, Wong T (2017). Community engagement in sexual health and uptake of HIV testing and syphilis testing among MSM in China: a cross-sectional online survey. J Int AIDS Soc.

[20] Tucker JD, Muessig KE, Cui R, Bien CH, Lo EJ, Lee R (2014). Organizational characteristics of HIV/syphilis testing services for men who have sex with men in South China: a social entrepreneurship analysis and implications for creating sustainable service models. BMC Infect Dis.

[21] Tucker J, Fenton K, Peckham R, Peeling R (2012). Social entrepreneurship for sexual health (SESH): a new approach for enabling delivery of sexual health services among most-at-risk populations. PLoS Med.

[22] Chen Y, Gong Y, Yang T, Song X, Li J, Gan Y (2016). Antimicrobial resistance in Neisseria gonorrhoeae in China: a meta-analysis. BMC Infect Dis.

[23] Yin YP, Han Y, Dai XQ, Zheng HP, Chen SC, Zhu BY (2018). Susceptibility of Neisseria gonorrhoeae to azithromycin and ceftriaxone in China: A retrospective study of national surveillance data from 2013 to 2016. PLoS Med.

[24] Kenyon CR, De Baetselier I, Crucitti T (2018). Does gonorrhoea screening intensity play a role in the early selection of antimicrobial resistance in men who have sex with men (MSM)? A comparative study of Belgium and the United Kingdom. F1000Res.

[25] Kenyon C, Laumen J, Van Dijck C (2020). Could Intensive Screening for Gonorrhea/Chlamydia in Preexposure Prophylaxis Cohorts Select for Resistance? Historical Lessons From a Mass Treatment Campaign in Greenland. Sex Transm Dis.

[26] Halpaap BM, Tucker JD, Mathanga D, Juban N, Awor P, Saravia NG (2020). Social innovation in global health: sparking location action. Lancet Glob Health.

[27] Hyde CR (1999). Pay It Forward.

[28] Murphy K. Ma'am, your burger has been paid for. The New York times. 2013. https://www.nytimes.com/2013/10/20/opinion/sunday/maam-your-burger-has-been-paid-for.html.

[29] Cueva M (2014). Thanks, au lait: 750 pay it forward at Starbucks location. CNNcom.

[30] Wegert T. JetBlue pays it forward through a social storytelling campaign with no end in sight. The content strategist. 2014. https://contently.com/2014/11/13/jetblue-pays-it-forward-through-a-social-storytelling-campaign-with-no-end-in-sight/.

[31] Jung MH, Nelson LD, Gneezy A, Gneezy U (2014). Paying more when paying for others. J Pers Soc Psychol.

[32] Li KT, Tang W, Wu D, Huang W, Wu F, Lee A (2019). Pay-it-forward strategy to enhance uptake of dual gonorrhea and chlamydia testing among men who have sex with men in China: a pragmatic, quasi-experimental study. Lancet Infect Dis.

[33] Yang F, Zhang TP, Tang W, Ong JJ, Alexander M, Forastiere L, et al. Pay-it-forward gonorrhea and chlamydia testing among men who have sex with men in China: a randomized controlled trial. Lancet Infect Dis. 2020;20(8):976–82. 10.1016/S1473-3099(20)30172-9 PMC8957706 32530426

[34] US department of health and human services. Principles of community engagement. second edition.: ATSDR; 2015. Available from: https://www.atsdr.cdc.gov/communityengagement/pdf/PCE_Report_508_FINAL.pdf.

[35] Curran GM, Bauer M, Mittman B, Pyne JM, Stetler C (2012). Effectiveness-implementation hybrid designs: combining elements of clinical effectiveness and implementation research to enhance public health impact. Med Care.

[36] Mauss M. The Gift: Princeton University Press; 1925.

[37] Yan Y. The flow of gifts : reciprocity and social networks in a Chinese village. Stanford, Calif.: Stanford University Press; 1996. vi, 278 p. p.

[38] Nowak MA, Roch S (2007). Upstream reciprocity and the evolution of gratitude. Proc Biol Sci.

[39] Wallerstein NB, Duran B (2006). Using community-based participatory research to address health disparities. Health Promot Pract.

[40] Stark E, Ali D, Ayre A, Schneider N, Parveen S, Marais K (2020). Coproduction with Autistic Adults: Reflections from the Authentistic Research Collective. Autism in Adulthood.

[41] O'Mara-Eves A, Brunton G, Oliver S, Kavanagh J, Jamal F, Thomas J (2015). The effectiveness of community engagement in public health interventions for disadvantaged groups: a meta-analysis. BMC Public Health.

[42] Erku D, Khatri R, Endalamaw A, Wolka E, Nigatu F, Zewdie A (2023). Community engagement initiatives in primary health care to achieve universal health coverage: A realist synthesis of scoping review. PLoS ONE.

[43] WHO. Community engagement: a health promotion guide for universal health coverage in the hands of the people. Geneva; 2020. https://www.who.int/publications/i/item/9789240010529.

[44] Chiong R, Kirley M (2015). Promotion of cooperation in social dilemma games via generalised indirect reciprocity. Connect Sci.

[45] Nowak MA, Roch S (2007). Upstream reciprocity and the evolution of gratitude. Proc Biol Sci.

[46] WHO/TDR/SESH/SIHI (2018). Crowdsourcing in Health and Health Research: A Practical Guide.

[47] Han L, Tang W, Ritchwood TD, Day S, Wei S, Bao H, et al. Joint international consensus statement on crowdsourcing challenge contests in health and medicine: results of a modified delphi process. BMJ Open. 2021;11:e048699. 10.1136/bmjopen-2021-048699 PMC8573649 34740928

[48] Hussey MA, Hughes JP (2007). Design and analysis of stepped wedge cluster randomized trials. Contemp Clin Trials.

[49] Sung A, Zhang TP, Huang W, Tang W, Alexander M, Forastiere L (2022). Development of a Psychometric Tool to Measure Community Solidarity Among Sexual Minorities: Evidence From a Pay-it-Forward Randomized Controlled Trial. Sex Transm Dis.

[50] Zak PJ, Kurzban R, Matzner WT (2005). Oxytocin is associated with human trustworthiness. Horm Behav.

[51] Kosfeld M, Heinrichs M, Zak PJ, Fischbacher U, Fehr E (2005). Oxytocin increases trust in humans. Nature.

[52] Barraza JA, McCullough ME, Ahmadi S, Zak PJ (2011). Oxytocin infusion increases charitable donations regardless of monetary resources. Horm Behav.

[53] Glasgow RE, Vogt TM, Boles SM (1999). Evaluating the public health impact of health promotion interventions: the RE-AIM framework. Am J Public Health.

[54] Sweetman D, Badiee M, Creswell JW (2010). Use of the Transformative Framework in Mixed Methods Studies. Qual Inq.

[55] Jackson KM, Pukys S, Castro A, Hermosura L, Mendez J, Vohra-Gupta S (2018). Using the transformative paradigm to conduct a mixed methods needs assessment of a marginalized community: Methodological lessons and implications. Eval Program Plann.

